# Focal radiotherapy boost to MR-visible tumor for prostate cancer: a systematic review

**DOI:** 10.1007/s00345-023-04745-w

**Published:** 2024-01-20

**Authors:** Anna M. Dornisch, Allison Y. Zhong, Darren M. C. Poon, Alison C. Tree, Tyler M. Seibert

**Affiliations:** 1 Department of Radiation Medicine and Applied Sciences, UC San Diego School of Medicine, La Jolla, CA USA; 2 University of California San Diego School of Medicine, La Jolla, CA USA; 3 Comprehensive Oncology Centre, Hong Kong Sanatorium and Hospital, Happy Valley, Hong Kong, Special Administrative Region of China; 4 The Royal Marsden NHS Foundation Trust, Sutton, UK; 5 Division of Radiotherapy and Imaging, Institute of Cancer Research, Sutton, UK; 6 Department of Bioengineering, UC San Diego Jacobs School of Engineering, La Jolla, CA USA; 7 Department of Radiology, UC San Diego School of Medicine, La Jolla, CA USA

**Keywords:** Focal boost, Localized prostate cancer, Magnetic resonance imaging, Intraprostatic lesion, External beam radiation therapy

## Abstract

**Purpose:**

The FLAME trial provides strong evidence that MR-guided external beam radiation therapy (EBRT) focal boost for localized prostate cancer increases biochemical disease-free survival (bDFS) without increasing toxicity. Yet, there are many barriers to implementation of focal boost. Our objectives are to systemically review clinical outcomes for MR-guided EBRT focal boost and to consider approaches to increase implementation of this technique.

**Methods:**

We conducted literature searches in four databases according to the Preferred Reporting Items for Systematic Reviews and Meta-Analysis guideline. We included prospective phase II/III trials of patients with localized prostate cancer underdoing definitive EBRT with MR-guided focal boost. The outcomes of interest were bDFS and acute/late gastrointestinal and genitourinary toxicity.

**Results:**

Seven studies were included. All studies had a median follow-up of greater than 4 years. There were heterogeneities in fractionation, treatment planning, and delivery. Studies demonstrated effectiveness, feasibility, and tolerability of focal boost. Based on the Phoenix criteria for biochemical recurrence, the reported 5-year biochemical recurrence-free survival rates ranged 69.7–100% across included studies. All studies reported good safety profiles. The reported ranges of acute/late grade 3 + gastrointestinal toxicities were 0%/1–10%. The reported ranges of acute/late grade 3 + genitourinary toxicities were 0–13%/0–5.6%.

**Conclusions:**

There is strong evidence that it is possible to improve oncologic outcomes without substantially increasing toxicity through MR-guided focal boost, at least in the setting of a 35-fraction radiotherapy regimen. Barriers to clinical practice implementation are addressable through additional investigation and new technologies.

## Introduction

Standard radiation therapy (RT) for prostate cancer treats the entire prostate to approximately the same dose. Dose escalation of RT to the whole prostate improves biochemical disease-free survival(bDFS) [1] but comes at the expense of increased late toxicity [1, 2]. This has spurred efforts to advance radiation therapy delivery to maximize disease control while minimizing toxicity. Radiation dose to normal tissues (and the volume of the normal tissue receiving a given dose) is usually directly related to risk of toxicity [3]. One plausible way to minimize the risk of toxicity is to limit the volume of tissue treated with high-dose RT. Local recurrence of prostate cancer usually occurs at the same site as the dominant primary tumor at baseline [4], and histopathologic data confirmed that clinically significant local recurrence after radiation therapy typically occurs at the site of the primary tumor [5]. Therefore, there is interest in escalating treatment of dominant intraprostatic lesions (IPLs), while maintaining acceptable doses to the whole prostate as a rational approach to enhancing the therapeutic ratio and local tumor control.

MR is the current imaging method of choice for detection and local staging of prostate cancer. While MR has been incorporated into radiation treatment planning for decades, early utilization of MR focused on delineating the prostate to reduce the amount of irradiated rectal tissue [6]. As MR has become more accurate in identifying intraprostatic tumors, researchers and clinicians have begun using MR for target delineation of dose escalation [7, 8]. Meanwhile, the Prostate Imaging Reporting and Data System (PI-RADS) guidelines and subsequent updates [9] have standardized multiparametric MR for initial detection of prostate cancer to include T2-weighted (T2W) MR, diffusion-weighted imaging (DWI), and dynamic contrast-enhancing MR (DCE-MR). The PI-RADS guidelines are expressly for informing biopsy decisions, though their prominent clinical use for diagnostic radiology impacts other clinical uses of MR, such as RT planning.

In 2021, Kerkmeijer et al. published the results of the FLAME trial, a randomized phase III trial that demonstrated the addition of a focal RT boost to the MR-visible tumor improved bDFS for patients with localized intermediate and high-risk prostate cancer—without increased toxicity or decreased quality of life [10]. A recent meta-analysis by Poon et al. synthesized patient-level data from 17 prospective studies (through 2021) to assess the efficacy/safety of MR-guided external beam focal boost to IPLs [11]. The synthesized bDFS was 92.4% (95% CI 84.5–97.7%) for studies with follow-up greater than 5 years. Since then, there have been increasingly mature results published as well as ongoing clinical trials about focal boosts in a variety of radiation therapy schedules, including hypofractionation [12] and ultra-hypofractionation (also called stereotactic body radiation therapy, or SBRT, and stereotactic ablative body radiotherapy, or SABR) [13].

Our first aim was to synthesize the evidence supporting MR-guided focal boost to IPLs by reviewing relevant prospective studies. Our second aim was to discuss the barriers to implementation of focal boost and offer some approaches to address these barriers with the goal of increasing the clinical implementation of MR-guided focal boost.

## Methods

### Search strategy

The Preferred Reporting Items for Systematic Reviews and Meta-Analyses (PRISMA) guideline was followed. Four databases were searched from inception to June 1, 2023: PubMed, Cochrane, Embase, and Web of Science. The reference sections of all studies that were eventually selected for full-text review were examined for additional studies. Databases were searched and returned 66 articles from PubMed, 14 articles from Cochrane, 164 articles from Embase, and 29 articles from Web of Science. All abstracts were imported into EndNote 21 and references were screened for duplicates. After removal of duplicates, 225 citations remained. The non-duplicate abstracts were screened, applying inclusion and exclusion criteria by one reviewer (AD). After applying the criteria, 189 were excluded and 36 articles remained. Full texts were retrieved for these articles and the full texts were screened, applying inclusion and exclusion criteria. After screening, 29 articles were removed and 8 articles remained for data extraction.

### Study selection

The Population, Intervention, Comparator, Outcome and Study Design method was used to define literature inclusion criteria. The inclusion criteria for studies were a population comprised of patients with previously untreated localized prostate cancer undergoing definitive EBRT with MR-guided focal boost to IPLs. The outcome of interest was bDFS as well as acute/late toxicities. Prospective trials with > 10 patients enrolled were eligible. Trials with a median follow-up time of 36 months were eligible. An English version of the study must be available and only published studies were included.

The exclusion criteria were: (1) non-human studies; (2) retrospective data/analyses; (3) Phase I clinical trials; (4) studies involving patients with metastatic prostate cancer; (5) studies involving patients undergoing radiation other than EBRT; (6) studies using imaging modalities other than MR to delineate IPLs; (7) publications not in English; and (8) books, conference abstracts, and case reports. We examined the references of relevant reviews to identify extra studies for inclusion.

### Data extraction

Data were extracted and reviewed. Oxford Centre for Evidence-Based Medicine 2011 levels of evidence were assigned to each study. Comprehensive data were extracted and cross-checked, including the study and patient characteristics, treatment planning/delivery, and clinical outcomes.

## Results

**Fig. 1:**
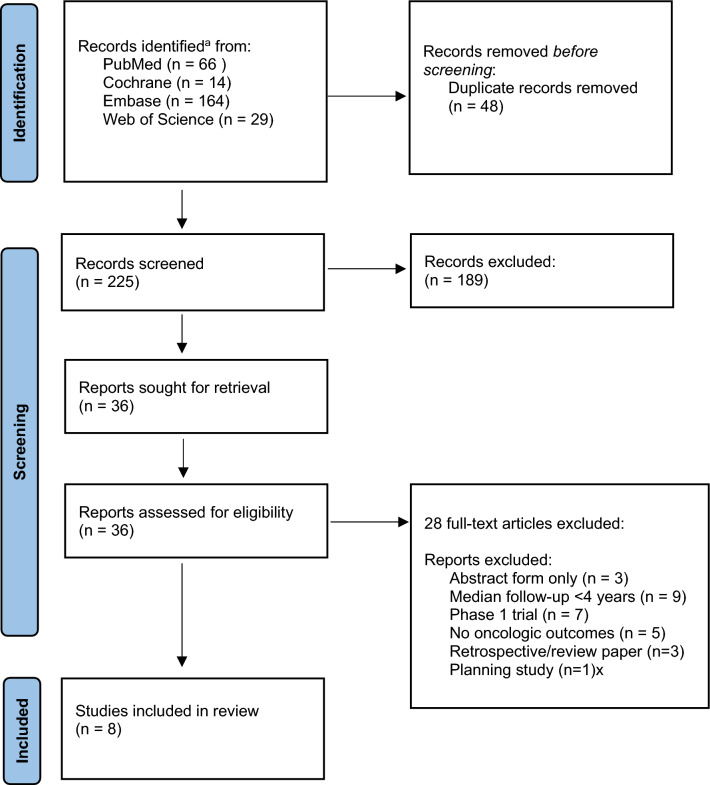
Preferred Reporting Items for Systematic Reviews and Meta-Analyses (PRISMA) flow diagram. ^a^Search Strategy: (((prostate cancer) or (prostate)) AND ((radiation therapy) OR (radiotherapy) OR (focal boost) OR (boost) NOT (brachytherapy)) AND ((intraprostatic lesion) OR (intraprostatic nodule) OR (dominant) OR (IPL) OR (IPN) OR (DIL)) AND ((Phase I Clinical Trial) OR (Phase II Clinical Trial) OR (Phase III Clinical Trial) OR (Phase IV Clinical Trial) OR (Controlled Clinical Trial) OR (Multicenter Study) OR (Randomized Controlled Trial) OR (Pragmatic Clinical Trial) OR (Comparative Study)))

The literature search yielded 225 publication records for screening after removal of duplicates. After screening, 36 full-text articles were assessed for eligibility. Of these, 28 were excluded; 7 prospective phase II/III trials, encompassing 8 publications, were ultimately included. Figure 1 shows the PRISMA flow diagram.

### Study characteristics

In the seven included studies (Table 1), 723 patients underwent MR-guided EBRT focal boost to IPLs. There were six phase II trials and one randomized phase III trial. Four studies included patients with low risk (LR), intermediate risk (IR), and high risk (HR). Two studies included patients only with LR and IR. The remaining studies included both IR and HR patients. Five studies presented at least 5 years of follow-up. All studies reported bDFS. All studies reported physician-measured toxicity outcomes and five studies reported patient-reported outcomes.

**Table 1:** Characteristics of the studies included in the review

(a) Study	Phase	Level evidence	Median follow-up, months (range)	Patients (*n*)	Risk group, *n* (%)			ADT (%)	Outcomes			
					Low	Intermediate	High		Toxicity	PRO	BFS	Other oncologic outcomes
Miralbell 2010 [7]	2	2	63 (18–88)	50	5 (10)	12 (24)	33 (66)	66	Yes	No	Yes	Yes
Buwenge 2020 [8]	2	2	120 (25–150)	44	6 (13.6)	18 (40.9)	20 (45.5)	100	Yes	No	Yes	Yes
FLAME Kerkmeijer 2021 [10] and Groen 2022 [14]	3	1	72 (58–86)	Focal Boost: 284 Standard: 287	2 (1)	43 (15)	239 (84)	60	Yes	Yes	Yes	Yes
2SMART Ong 2023 [16]	2	2	44 (NR)	30	2 (7)	28 (93)	0	7	Yes	Yes	Yes	Yes
Cloitre 2023 [15]	2	2	61 (42–110)	33	1 (3)	14 (42)	18 (55)	3	Yes	Yes	Yes	No
Maas 2023 [17]	2	2	59.5 (11–86)	26	6 (8)	20 (92)	0	27	Yes	Yes	Yes	No
DELINEATE Tree 2023 [12]	2	2	> 60 (NR)	256	0	111 (43)	145 (57)	100	Yes	Yes	Yes	No

(b) Study	Treatment planning characteristics						Toxicity outcomes							
	Dose (Gy)	Fractionation	Boost type	IPL Boost Dose (Gy)	GTV_IPL_-PTV_IPL_ margins (mm)	Urethra Dose constraint	Acute (% of patients)				Late (% of patients)			
							GU		GI		GU		GI	
							G2 +	G3 +	G2 +	G3 +	G2 +	G3 +	G2 +	G3 +
Miralbell 2010 [7]	64	32	Sequential—3dCF followed by IMRT boost	74–80	NR	Yes—Not Reported	50	13	8	0	12	0	20	10
Buwenge 2020 [8]	72	40	SIB	80	15: GTV_IPL_ + 5 = CTV_IPL_ + 10 = PTV_IPL_	NR	NR	NR	NR	NR	NR	4.5	NR	2.3
FLAME Kerkmeijer 2021 [10] & Groen 2022 [14]	77	35	SIB	95	0	None	NR	NR	NR	NR	27.8	5.6	12.7	1.4
2SMART Ong 2023 [16]	26	2	SIB	32	0	Yes—Not Reported	56	0	3	0	50	0	10	0
Cloitre 2023 [15]	36.25	5	SIB	45–50	3	D_1cc_ < 39 Gy D_0.1 cc_ < 41 Gy	15	0	6	0	12	0	3	0
Maas 2023 [17]	36.25	5	SIB	40	0–5	Dmax < 38.78 Gy	42	0	4	0	39	0	11.5	0
DELINEATE Tree 2023 [12]	A: 74 B: 60 C: 74	A: 37 B: 20 C: 37	SIB	A: 82 B: 67 C: 82	2	A: D_2%_ < 83 Gy B: D_2%_ < 67.3 Gy C: D_2%_ < 83 Gy	A: 38 B: 36 C: 38	NR	A: 11 B: 14 C: 15	NR	A: 13 B: 18 C:18	A: 4 B: 2 C: 3	A: 13 B: 15 C: 21	A: 0 B: 1 C: 3

### Treatment planning/delivery

#### MR usage

All studies utilized MR to delineate IPLs. Two studies utilized MR with endorectal coil [7, 8]. Four studies required use of multiparametric MR [10, 12, 14–16]. All studies except for 2SMART described the sequences used to delineate IPLs; they utilized at least *T*_*2*_-weighted imaging (T2W). Three studies utilized at least T2W and DWI [10, 12, 14, 17]. DWI generally includes generation of ADC maps, where specifically cited in one study [17] and are the primary images for most lesions according to PI-RADS radiologist guidelines [9]. The FLAME trial utilized the following sequences: T2W, DWI, and DCE. Maas et al. utilized the following sequences: T2W, DWI, and ADC. Diagnostic MRs were usually fused (registered) with planning CTs to aid in contouring but one study displayed them side-by-side [12]. Many studies did not explicitly characterize IPL criteria for focal boost eligibility.

#### Simulation

All studies used CT simulation. Most studies did not clarify the temporal relationship between CT simulation and planning MR; the lone exception, DELINEATE, performed CT and MR on the same day [12]. All but one study described techniques used for verification of patient setup [8]. Five studies required gold fiducial placement at simulation [10, 12, 14–17]. One study used infrared external markers [7]. All studies used bladder and rectum control. Most commonly, patients were scanned with full bladder and empty rectum. One study treated patients with empty bladder [7].

#### Technique/fractionation

Six studies treated patients using IMRT/VMAT; these studies utilized a simultaneous integrated boost approach [8, 10, 12, 14–17]. One study treated patients with a sequential boost and utilized 3D conformal radiation therapy for the initial course followed by IMRT boost [7]. Three studies treated patients with standard fractionation [7, 8, 10, 14]. One study had separate arms that treated patients with standard fractionation and moderate hypofractionation [12]. There were three SBRT trials with two studies investigating five-fraction SBRT [15, 17] and one trial studying two-fraction SBRT [16].

#### Volumes

All IMRT/VMAT studies defined the gross tumor volume (GTV) as the IPL (GTV_IPL_), delineated by planning MR. The prostate clinical target volume (CTV) was the whole prostate plus proximal seminal vesicles. Two studies included elective nodal irradiation [7, 12]. The GTV-CTV boost expansion was not reported in all studies and ranged from 0 to 5 mm. The CTV-planning target volume (PTV) margin was not reported in all studies. Most studies defined a PTV_IPL_ as the PTV of the focal boost and a PTV_p_ as the PTV encompassing the prostate and seminal vesicles. PTV expansions were not reported in all studies; of those reported, they were highly variable. GTV_IPL_ to PTV_IPL_ expansion ranged from 0 to 5 mm. CTV to PTV_p_ expansions ranged from 2 to 10 mm. These expansions were mostly isotropic with a smaller margin posteriorly.

#### Dose

Only four studies reported the assumed alpha/beta ratio for dose planning; this ranged from 1.2 to 3 [7, 10, 12, 14, 16]. The dose (Gy) to PTV_p_ was as follows: 64–77 Gy for standard fractionation, 60 Gy for moderate hypofractionation, 36.25 Gy for 5-fraction SBRT, and 26 Gy for 2-fraction SBRT. The dose (Gy) to PTV_IPL_ was as follows: 74–95 Gy for standard fractionation; 67 Gy for moderate hypofractionation; 40–50 Gy for 5-fraction SBRT, and 32 Gy for 2-fraction SBRT.

#### OARs

The number of concerning organs at risk (OARs), their contouring guidelines, and dose constraints varied widely among studies. All studies included the rectum and bladder. Urethra-sparing was variable. The FLAME trial did not have a urethral dose constraint. Four trials included a urethral dose constraint [12, 15–17]; one arm of DELINEATE utilized a foley catheter to contour the urethra, while all other arms/trials contoured based on MR/CT simulation. Spacer devices were required for two of the SBRT trials [15, 16] as well as the sequential IMRT boost trial [7].

### Outcomes

#### Toxicity

All studies included physician-reported toxicities. Five studies reported on acute GI and GU toxicity rates, usually defined as occurring < 90 days after the completion of treatment (Table 2a) [7, 12, 15–17]. All studies provided late GI and GU toxicity rates (Table 2b). Five studies included patient-reported outcomes data on quality of life, including urinary, bowel, and sexual function [10, 12, 14–17]. Additionally, two studies (hypo-FLAME and hypo-FLAME 2.0) have recently published acute toxicity outcomes. Hypo-FLAME reported acute grade 2 GU and GI toxicity rates of 34% and 5%, respectively, with five-fraction SBRT delivered weekly [18]. Hypo-FLAME 2.0 showed acute grade 2 + GU and GI toxicity rates of 47.5% and 7.4% with five-fraction SBRT delivered biweekly [13].

**Table 2:** Range of percentage of patients experiencing physician-reported acute (a) and late (b) gastrointestinal and genitourinary toxicities across included studies

	GI toxicity (%)	GU toxicity (%)
(a)		
Grade 2 +	3–8%	15–56%
Grade 3 +	0%	0–13%
(b)		
Grade 2 +	3–20%	12–50%
Grade 3 +	1–10%	0–5.6%

#### Biochemical outcomes

All studies reported bDFS, which ranged from 69.7% to 100%. All studies defined biochemical failure with the Phoenix definition (PSA nadir + 2 ng/mL). The FLAME trial provided level 1 evidence for focal boost. In the FLAME trial, the 5-year bDFS in the focal boost arm was significantly higher (92% versus 85%; hazard ratio 0.45, 95% CI 0.28–0.71; p < 0.001) than in the standard arm without focal boost [10].

#### Other oncologic outcomes

There was significant heterogeneity of reporting of other oncologic outcomes. Overall survival was explicitly reported in several studies; Buwenge et al. reported 5-year and 10-year OS of 95.5% and 87.8%, respectively [8]. Disease-specific survival was also reported in several studies. Miralbell et al. reported 100% disease-specific survival at 5 years [7]. FLAME showed significantly improved disease-free survival in the focal boost arm at up to 7 years of follow-up [10, 14]. Metastasis-free survival (MFS) was also explicitly reported in several studies. Buwenge et al. reported 5-year MFS of 100% and 10-year MFS of 97.6% [8]. Significantly, in a patterns of failure report from FLAME, focal boosting significantly decreased local failure (HR 0.33, 95% CI 0.14–0.78) and regional and distant MFS (HR 0.58, CI 0.35–0.93) [14].

## Discussion

All studies demonstrated efficacy, safety, tolerability, and feasibility of MR-delineated EBRT focal boost. The FLAME trial provided strong evidence that focal boost to tumors visible on MR improves outcomes for prostate cancer patients without significant increase in toxicity or detriment to quality of life. Despite this, there are many barriers that exist to widespread adoption into routine clinical practice. Recently, a global survey of radiation oncologists highlighted five barriers to adoption: (1) not being aware or convinced of the benefit of focal boost, (2) concerns about risk of additional toxicity, (3) concerns about registration accuracy between MR and CT, (4) concerns about tumor delineation/comfort with MR, and (5) concerns about planning [43]. Assessing the current practice patterns of treating radiation oncologists and their hesitancies about focal boosting will guide further research and concentrate efforts to increase utilization of focal boost.

Here, we offer approaches to address these barriers.

### Barrier #1: not aware or not convinced of benefit

In this systematic review, we summarized results of phase II/III trials that have looked at the safety and efficacy of MR-guided focal boost. Additionally, Poon et al. demonstrated in a meta-analysis of 17 prospective clinical trials, including FLAME, that biochemical disease-free survival was 95.0% with acceptable toxicity profile. There are more data coming from PIVOTALBoost, a 4-arm Phase III randomized control trial looking at prostate and pelvis versus prostate alone radiotherapy with or without prostate boost [19]. Importantly, the FLAME trial provided strong evidence that a focal boost to tumors increased bDFS without impacting toxicity and quality of life. However, hard endpoints, like metastasis-free survival are likely critical. Although the primary outcome for FLAME was bDFS, an updated publication showed that focal boosting decreased local failure (hazard ratio 0.33, 95% CI 0.14–0.78) and improved *regional and distant* metastasis-free survival (HR 0.58, 95% CI 0.35–0.93) [14]. Currently, no prostate-cancer-specific mortality benefit, distant metastasis-free survival benefit, or overall survival benefit has been demonstrated. At minimum in the setting of standard or nearly standard fractionation (FLAME used 2.2 Gy per fraction to the whole prostate), there may be a role for increasing physician awareness of the updated FLAME results. Importantly, further research efforts should distinguish between not aware of demonstrated benefit versus not convinced of sufficient benefit as these barriers require different strategies to overcome.

### Barrier #2: concerns about MR–CT registration/planning

We identified concerns around MR–CT registration and treatment planning as workflow barriers to adoption [43]. Several institutions have developed an MR-only workflow and simulation [20–22], which eliminates need for MR–CT registration. The MR-PROTECT trial showed the feasibility of an MR-only prostate radiotherapy workflow [23]. However, generating radiotherapy plans based only on predicted electron density from MR, rather than using measured Hounsfield units from a CT simulation, has not been adopted at most radiotherapy centers. A second approach is MR simulation, where both CT and MR simulation are acquired for each patient (i.e., both scans with patients in treatment position, using the same custom immobilization devices, and with consistent bowel/bladder preparation instructions). These efforts to minimize differences in patient positioning might improve reliability of image registration. A more common clinical situation, though, is registering a diagnostic MR with simulation CT, which is often temporally spaced and performed with different patient positioning. Further complicating registration, the patient may have started androgen deprivation therapy between diagnostic MR and CT simulation, leading to substantial shrinkage of the prostate. Similarly, placement of a rectal spacer changes the internal anatomy, complicating MR–CT registration if both scans are not done after spacer placement.

There are various strategies for registration, including box-based [24], and local registration, but these are time intensive and require a degree of expertise. In contrast, various deformable registration tools exist that could automize the registration process. Specifically, Ciardo et al. reported a robust and accurate methodology to transfer information from diagnostic MR to planning CT, and Fu et al. are developing a deep learning network to accurate register the prostate on MR to CBCT [25, 26]. These are prostate-specific tools that would standardize registration with the aim of minimizing the variability of registration, as well as the time and effort required to implement. Placement of fiducials can also help with registration of the prostate, itself, and was part of the FLAME protocol [10]. In addition to utilizing a static MR in treatment planning for focal boost, MR-Linac could allow for online adaptive target delineation of IPLs at treatment for focal boosting. Providing training to dosimetrists and using knowledge-based planning will likely reduce planning concerns [27, 28]. Automated planning tools for focal prostate boost are also being developed [29].

### Barrier #3: tumor delineation/comfort for MR

Paralleling concerns about MR–CT registration, tumor delineation, and comfort using MR are substantial barriers to implementation [43]. MR-guided boost is not the only prostate radiotherapy modality improved by MR. The prostate is better visualized /delineated on MR than CT. The MIRAGE trial showed that MR-guided prostate SBRT significantly reduced moderate acute physician-scored toxic effects and decrements in patient-reported quality of life compared to CT-guided SBRT [30]. Van Schie et al. demonstrated considerably different interpretations of multiparametric MR in tumor bed contouring between institutions in FLAME [31]; even without contouring guidelines and some sub-optimal volumes, FLAME still showed a clinical improvement to focal boost. Therefore, there are multiple reasons for radiation oncologists to use MR for targeting/planning purposes when treating prostate cancer patients.

However, there needs to be radiation oncology-specific training in prostate MR interpretation given the steep learning curve for interpretation of prostate MR, even for diagnostic radiologists. For example, the accuracy of detecting tumors in the peripheral zone, tumors in the transitional zone, and ECE by diagnostic radiology fellows significantly improved after a dedicated didactic training program [32]. In addition to dedicated education, involving diagnostic radiologists in planning would be helpful. This could take many variations, from annotated imaging to video conferencing during contouring.

Simultaneously, there is a need for studies to determine the optimal target on MR. The ReIGNITE RT Boost trial showed that radiation oncologists can struggle to correctly contour boost targets even when given a detailed written description of the lesion [33]. ReIGNITE also investigated whether radiation oncologists’ contouring accuracy would be improved if they were given advanced MR images created using a technique called Restriction Spectrum Imaging (RSI). RSI restriction score (RSIrs) maps were previously shown to be more specific for clinically significant prostate cancer [34–36]. Without any other educational intervention or training, when radiation oncologists used the RSIrs maps, their contouring reliability and accuracy improved markedly [33].

The value of PSMA-PET for focal boost target delineation is also under investigation. The HypoFocal Phase II trial presented their 6-month planned safety analysis data, which appears promising [37]. We found no other Phase II/III trials yet published using this approach; retrospective studies suggest the technique is of interest and will likely be useful as an MR alternative or complement [38–41].

### Barrier #4: toxicity concerns

Radiation oncologists cited concerns about increased toxicity [43]. The FLAME trial reported no increased toxicity at median follow-up of six years. Subsequently, Groen et al. modeled normal tissue complication probability curves using FLAME and concluded that increasing dose to the bladder and urethra will result in a significant increase in GU toxicity [42]. The authors recommended a urethral dose constraint, which was then incorporated into hypo-FLAME [18]. Further questions have risen regarding the safety of focal boost with various fractionation schemes beyond the 35-fraction approach used in FLAME. We reviewed several ongoing phase II trials looking at the role of focal boost in moderate hypofractionation and ultra-hypofractionation schedules; with the maturation of these studies, we can expect additional toxicity data. More definitive guidelines incorporating treatment planning parameters—such as appropriate dose targets, dose constraints, contouring, and margins—might also facilitate adoption of the focal boost technique. Consensus dose equivalents for focal boost using hypofractionated regimens would likely also be helpful. Lastly, the role of spacers in focal boost deserves exploration.

### Brachytherapy

Although this review focuses only on focal external beam dose escalation with MR guidance, brachytherapy is another studied strategy for dose escalation. In a survey of radiation oncologists who treat prostate cancer, 14% of 258 respondents indicated that they prefer brachytherapy boost to focal boost with external beam [43]. The ASCENDE-RT trial reported that addition of brachytherapy boost significantly improves time to biochemical progression, though at the expense of significantly increased acute and late GU toxicity [44, 45]. The ongoing PIVOTALBoost trial may provide additional insight on the benefits and risks of brachytherapy as a boost strategy. PIVOTALBoost allows brachytherapy or focal external beam for participants randomized to the boost arm, so the trial may also provide further insight into the preferences and comfort of radiation oncologists with each strategy. In the abovementioned survey, 20% of radiation oncologists who only treat genitourinary cancers indicated a preference for brachytherapy, compared to 9% of generalists [43]. For oncologists not offering brachytherapy (e.g., due to toxicity concerns, technical challenges, or lack of resources), external focal boost may represent a more feasible strategy, though this remains to be seen.

## Limitations

A limitation of the available literature on this emerging topic is that there is only one randomized phase III trial that directly evaluated focal RT boost. Additionally, many studies reviewed were small: 5 of 7 included studies reported on less than 50 patients each. Due to the natural history of prostate cancer, long-term follow-up data would be required to demonstrate an overall survival or distant-metastasis-free survival benefit. Nonetheless, at present, the lone phase III randomized trial (FLAME) demonstrated that a meaningful clinical benefit is achievable without increasing side effects for patients.

## Conclusion

We reviewed seven prospective phase II/III trials about MR-guided focal boost. FLAME showed that it is possible to improve oncologic outcomes without increasing toxicity. Despite this strong evidence, there are many barriers to implementation in clinical practice. These barriers are addressable through additional investigation and new technologies.

## Data Availability

Data availability is not applicable to this article as no new data were created or analyzed in this study.
